# Molecular basis of nitrogen starvation-induced leaf senescence

**DOI:** 10.3389/fpls.2022.1013304

**Published:** 2022-09-23

**Authors:** Yasuhito Sakuraba

**Affiliations:** Plant Functional Biotechnology, Biotechnology Research Center, The University of Tokyo, Tokyo, Japan

**Keywords:** leaf senescence, nitrogen (N), N starvation, N remobilization, transcriptional regulation

## Abstract

Nitrogen (N), a macronutrient, is often a limiting factor in plant growth, development, and productivity. To adapt to N-deficient environments, plants have developed elaborate N starvation responses. Under N-deficient conditions, older leaves exhibit yellowing, owing to the degradation of proteins and chlorophyll pigments in chloroplasts and subsequent N remobilization from older leaves to younger leaves and developing organs to sustain plant growth and productivity. In recent years, numerous studies have been conducted on N starvation-induced leaf senescence as one of the representative plant responses to N deficiency, revealing that leaf senescence induced by N deficiency is highly complex and intricately regulated at different levels, including transcriptional, post-transcriptional, post-translational and metabolic levels, by multiple genes and proteins. This review summarizes the current knowledge of the molecular mechanisms associated with N starvation-induced leaf senescence.

## Introduction

Leaf senescence, the final phase of leaf development, is a highly controlled process accompanied by massive transcriptional and metabolic changes that destabilize intracellular organelles and macromolecules and lead to the translocation of nutrients into developing tissues and storage organs. In the past two decades, molecular mechanisms underlying the regulation of leaf senescence have been extensively studied (Woo et al., 2019; Guo et al., 2021; Zhang et al., 2021). The initiation of leaf senescence is tightly controlled by internal factors, such as the state of phytohormones, photosynthesis, sugars, and other metabolites (Sakuraba et al., 2012a; Jibran et al., 2013; Woo et al., 2019), and external stimuli such as high salinity, drought, pathogens, and light (Quirino et al., 1999; Gepstein and Glick, 2013; Zhang et al., 2012; Sakuraba, 2021a). In addition to these external stimuli, the deficiency of mineral nutrients in the soil is known to cause premature leaf yellowing.

Nitrogen (N) is a key mineral nutrient for plants and a major constituent of molecules essential for plant growth, such as nucleic acids, amino acids, and chlorophyll (Marschner, 1995). Thus, the availability of N is often a limiting factor for many aspects of plant growth and development. In the natural ecosystem and the field, plants frequently encounter N deficiency and thus exhibit N deficiency responses to efficiently acquire and use available N in the soil (Kiba and Krapp, 2016). N deficiency responses include the modification of root architecture (Gruber et al., 2013) and the expression of genes associated with high-affinity transport systems for nitrate and ammonium (Lezhneva et al., 2014; Kiba and Krapp, 2016) to promote the uptake of N sources. In addition, leaf yellowing due to the remobilization of N sources from older leaves to younger leaves and reproductive organs is also one of the representative N deficiency responses and is considerably important for plants to sustain growth and productivity. On the other hand, N starvation-induced leaf yellowing in young seedlings causes severe growth defects (Sakuraba et al., 2021b). Therefore, understanding the molecular mechanisms underlying N starvation-induced leaf senescence is critical for establishing sustainable agriculture under N-deficient conditions.

In recent years, the regulatory mechanisms of N starvation-induced leaf senescence have been widely uncovered at the transcriptional, post-transcriptional, post-translational, and metabolic levels. This review summarizes the results of studies conducted to date on N starvation-induced leaf senescence in the model dicot *Arabidopsis thaliana* and in agronomically important crops.

## Metabolic changes in plants during N deficiency

Plants increase the capacity of N acquisition by enhancing root growth and upregulating the expression of genes encoding high-affinity nitrate and ammonium transporters under N deficiency stress (Kiba and Krapp, 2016). However, when these adaptations are not enough to provide a sufficient N supply, plants are forced to respond with further adaptive metabolic strategies that facilitate N remobilization to complete their life cycle.

The metabolome of Arabidopsis seedlings exposed to long-term N starvation showed dramatic changes (Krapp et al., 2011). Under N deficiency stress, the accumulation of nitrate and ammonium ions decreased rapidly. The total amino acid content of shoots gradually decreased during N starvation, while that of roots increased during the early phase of N starvation and then gradually returned to the level observed before the start of N starvation (Krapp et al., 2011). In shoots, the levels of N-rich amino acids such as glutamine (Gln), glutamate (Glu), asparagine (Asn), and aspartate (Asp) significantly decreased within a few days of N starvation; the accumulation of hydrophobic amino acids, such as leucine (Leu), isoleucine (Ile), and valine (Val), showed no significant change; and the levels of a few minor amino acids, such as lysine (Lys), arginine (Arg), and histidine (His), increased during long-term N starvation (Krapp et al., 2011). Since other N-containing compounds such as proteins and chlorophylls are synthesized from amino acids, the reduction in amino acid levels during long-term N starvation directly affects the accumulation of these compounds, leading to the promotion of leaf yellowing (Krapp et al., 2011; Balazadeh et al., 2014).

On the other hand, the content of soluble sugars such as sucrose, fructose, and galactose increased dramatically in Arabidopsis plants during N starvation (Krapp et al., 2011; Balazadeh et al., 2014). Several studies reported that sugars play an important role in the promotion of leaf senescence. Direct application of sucrose and glucose induced yellowing in *Xanthium pensylvanicum* leaf discs and Arabidopsis seedling leaves, respectively (Khudairi, 1970; Wingler et al., 2006). Moreover, genetic mutants and transgenic plants with altered sugar accumulation or sensing exhibited differences in the promotion of leaf senescence. Transgenic tomato plants overexpressing Arabidopsis *HEXOKINASE1* (*AtHXK1*) exhibited accelerated leaf senescence (Dai et al., 1999). On the other hand, an Arabidopsis deficient mutant of *MALTOSE EXCESS 1* (*MEX1*) exhibited a pale-green leaf phenotype and premature leaf senescence (Stettler et al., 2009). Thus, increased accumulation of soluble sugars in plants under N-deficient conditions may contribute to the promotion of leaf senescence.

N starvation also alters the accumulation of some organic acids. During N starvation, the levels of fumarate and succinate significantly increased, while those of aconitate and citrate decreased in the shoots of Arabidopsis seedlings (Krapp et al., 2011). While the accumulation of fumarate was shown to be closely associated with the accumulation of amino acids (Pracharoenwattana et al., 2010), the involvement of these organic acids in the promotion or inhibition of leaf senescence has not yet been investigated.

## N deficiency induces the degradation of N-containing compounds and remobilization of N in older leaves

Under N deficiency stress, N is remobilized from senescing leaves to developing tissues, such as young leaves and other sink organs, in the form of nitrate, ammonium, urea, amino acids, and short peptides, leading to the promotion of leaf yellowing in older leaves. This N remobilization is accompanied by increased proteolysis activity in older leaves (Hörtensteiner and Feller, 2002). In addition, chlorophyll content, which is directly associated with the amount of photosystem proteins, dramatically decreases under N-deficient conditions (Hanaoka et al., 2002). Section 3 summarizes the molecular mechanisms underlying the degradation of N-containing compounds and remobilization of N that occur during N deficiency stress.

### Degradation of chloroplast proteins under N deficiency stress

**Figure 1:**
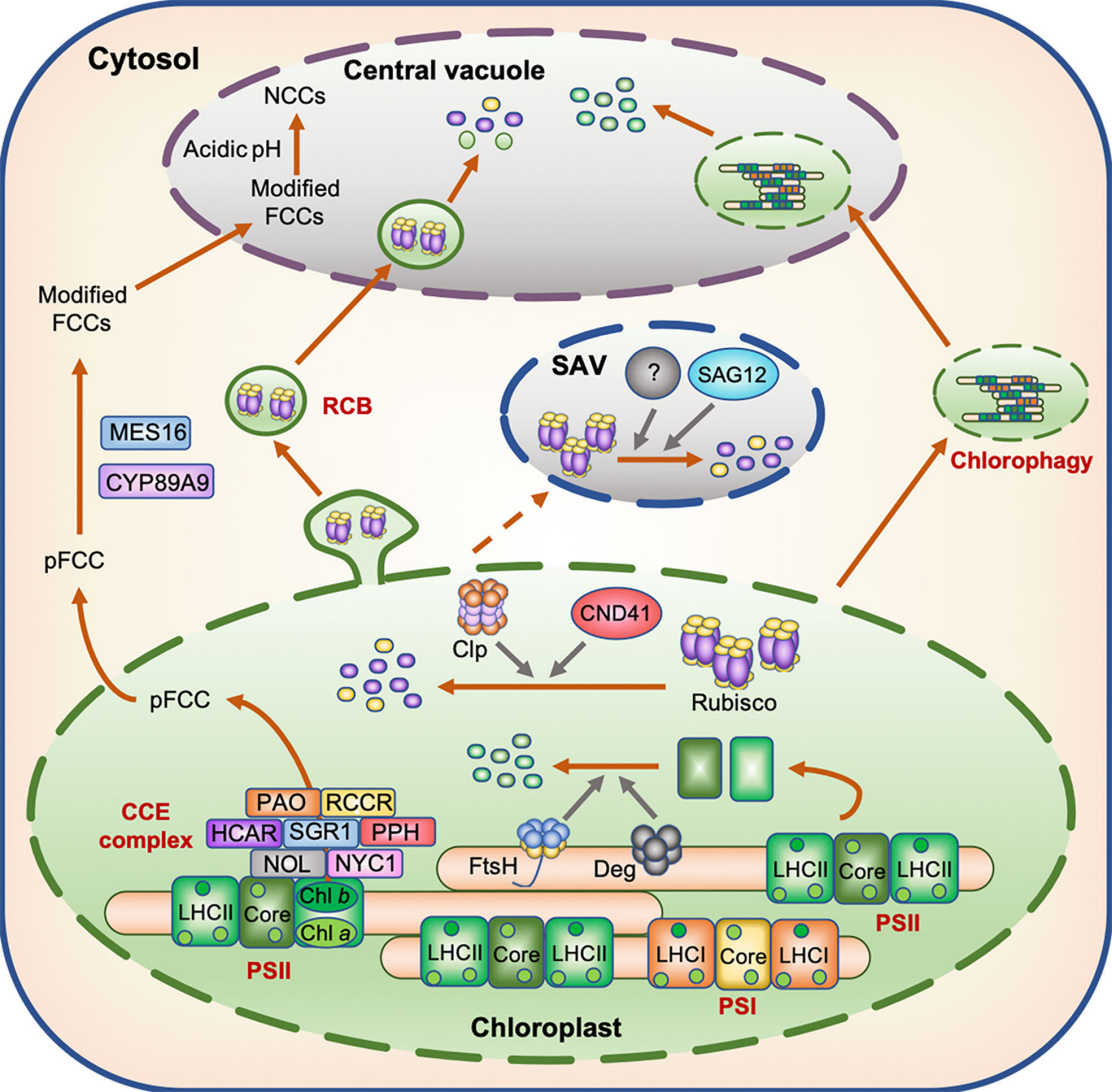
Model displaying the degradation of N-containing compounds in the chloroplast during leaf senescence. During leaf senescence, chloroplast proteins including Rubisco and photosystem subunits are believed to be degraded through several proteolytic processes mediated by proteases localized in the chloroplasts, senescence-associated vacuoles (SAVs), and central vacuoles as well as by Rubisco-containing bodies (RCBs) and chlorophagy. The degradation of chlorophyll molecules occurs in two distinct phases; the first phase is associated with the degradation of chlorophyll molecules by the chloroplast-localized chlorophyll catabolic enzyme (CCE) complex, while the second phase is associated with the translocation of colorless chlorophyll catabolites fluorescent chlorophyll catabolite (FCC) from chloroplasts to the central vacuole.

In the mesophyll cells of C3 plants, approximately 80% of N is located in chloroplasts, mainly as a component of ribulose 1,5-bisphosphate carboxylase/oxygenase (Rubisco; a stromal enzyme) and the light-harvesting complex (LHC; which contains chlorophyll pigments) (Peoples and Dalling, 1988; Makino et al., 2003). Previous studies showed that the accumulation of Rubisco dramatically decreased in the leaves of *Phaseolus vulgaris* (Crafts-Brandner et al., 1996) and Arabidopsis plants (Izumi et al., 2010) under N-deficient conditions. Rubisco and photosystem proteins are believed to be degraded under N deficiency stress through multiple proteolytic pathways, one of which is mediated by chloroplast proteases. Several studies reported the significance of chloroplast proteases in the degradation of photosystem proteins. For example, FtsH and DegP proteases are involved in the degradation of the damaged D1 protein (Lindahl et al., 2000; Haussühl et al., 2001), a core subunit of photosystem II (PSII). FtsH is also involved in the degradation of the Lhcb2 protein (Zelisko et al., 2005). Additionally, the chloroplast-localized aspartic protease CND41 was shown to mediate the degradation of Rubisco in tobacco leaves under N-deficient conditions (Kato et al., 2004). Although direct evidence is lacking, Rubisco is also speculated to be degraded by other stromal proteases, such as Clp (**Figure 1**). Indeed, Clp was shown to be involved in the degradation of Rubisco in the chloroplast of the green alga *Chlamydomonas reinhardtii* (Majeran et al., 2019).

Guiboileau and coworkers indicated the significance of autophagy in N remobilization under N deficiency stress. Tracer experiments using ^15^N-labeled nitrate showed that N remobilization into seeds was reduced in autophagy mutants under N deficiency stress (Guiboileau et al., 2012). Furthermore, the N content of rosette leaves was significantly higher in autophagy mutants than in the wild type (WT) (Guiboileau et al., 2013). Although the number of Rubisco-containing autophagosomes, called Rubisco-containing bodies (RCBs), decreased during the period of N deficiency (Izumi et al., 2010), a certain amount of Rubisco proteins is speculated to be translocated into the central vacuole through the autophagy pathway, and then degraded (**Figure 1**). In Arabidopsis plants subjected to dark-induced senescence, chlorophyll fluorescence was detected in the central vacuole of leaf cells (Wada et al., 2009), indicating that the macroautophagy system also functions in the transport of chlorophyll–apoprotein complexes and other thylakoid proteins from chloroplasts to the central vacuole during dark-induced leaf senescence and probably under other senescence-inducing conditions including N deficiency. Chlorophagy, in which whole chloroplasts are transported to the central vacuole (Nakamura and Izumi, 2018), may also be involved in the transport of thylakoid proteins to the central vacuole during leaf senescence (**Figure 1**).

While autophagy is certainly involved in N remobilization, its molecular mechanism remains to be elucidated. In previous studies, Arabidopsis autophagy mutants exhibited early leaf yellowing under N-deficient conditions (Thompson et al., 2005; Guiboileau et al., 2012) and during dark-induced leaf senescence (Thompson et al., 2005), and exhibited leaf necrosis under abiotic stresses such as high salinity and drought (Liu et al., 2009). Under these stress conditions, however, chlorophyll degradation should be impaired when autophagy operates properly, since autophagy is involved in the degradation of chloroplast proteins. On the other hand, the leaves of Arabidopsis autophagy mutants exhibited delayed leaf yellowing under mild abiotic stress conditions (Sakuraba et al., 2014a). This difference in the progression of chlorosis (or necrosis) of autophagy mutant leaves between severe and mild stress conditions may reflect the significance of autophagy in adapting to severe stress. It is possible that autophagy mutants cannot adapt to severe stress, since they cannot properly maintain their proteome balance under extremely unfavorable conditions and thus exhibit accelerated leaf yellowing and/or leaf necrosis. Investigation of the phenotype of autophagy mutants under different N concentrations will provide important insights into autophagy-mediated N remobilization that occurs under N deficiency stress.

During leaf senescence, senescence-associated vacuoles (SAVs), which show greater lytic activity than the central vacuole, are formed in the peripheral cytoplasm of mesophyll cells (Otegui et al., 2005). SAVs contain stromal proteins such as Rubisco and glutamine synthetase (GS) but do not contain thylakoid proteins such as D1, LHC of PSII (LHCII), and cytochrome *c* (Cyt *c*) (Martínez et al., 2008), indicating that SAVs are involved in the degradation of stromal proteins, but not thylakoid proteins during senescence. In wheat (*Triticum aestivum* L.), the activity of several vacuolar cysteine proteases increased in senescing leaves (Martínez et al., 2007). *SENESCENCE-ASSOCIATED GENE 12* (*SAG12*), which encodes a vacuolar cysteine protease, is one of the most widely used senescence marker genes. The expression of *SAG12* is strongly induced during leaf senescence (Lohman et al., 1994), and the encoded protein localizes to SAVs (Otegui et al., 2005). Therefore, SAG12 is thought to participate in the degradation of Rubisco proteins in SAVs (**Figure 1**). However, in the *sag12* knockout mutants of Arabidopsis, the degradation of Rubisco proteins was not affected under both high and low N conditions, whereas the activity of aspartic protease was greatly enhanced (James et al., 2018). These results suggest that some aspartic proteases compensate for the effect of *sag12* mutation on the degradation of Rubisco proteins. Thus, functional characterization of aspartic proteases in SAVs is necessary for further understanding the proteolytic process of stromal proteins in SAVs.

### Degradation of chlorophyll pigments under N-deficient conditions

**Figure 2:**
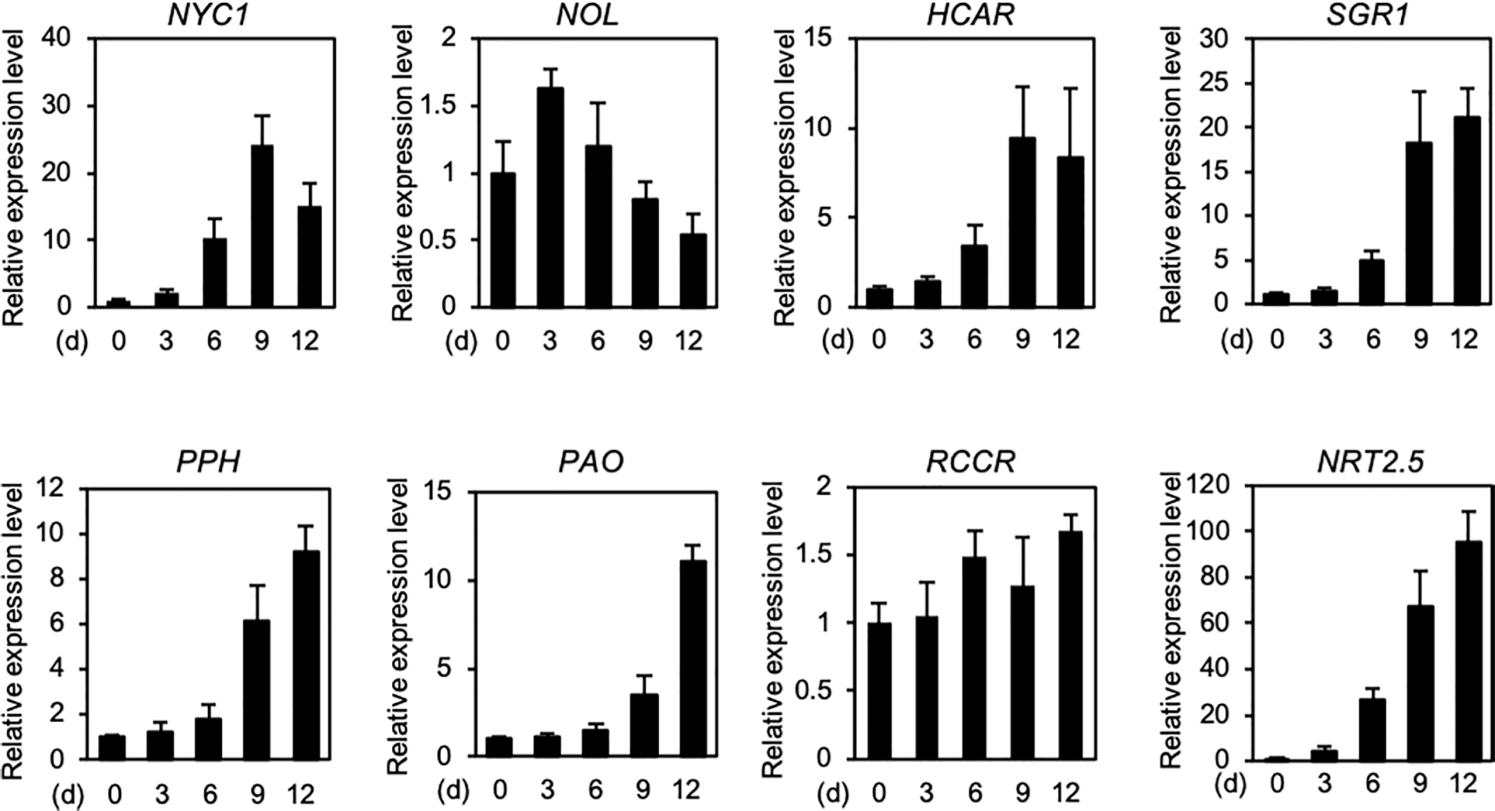
Expression profiles of seven genes encoding chlorophyll catabolic enzymes during N deficiency. Expression levels of seven chlorophyll catabolic enzyme-encoding genes *NYC1*, *NOL*, *HCAR*, *SGR1*, *PPH*, *PAO*, and *RCCR*, and a high-affinity nitrate transporter-encoding gene *NRT2.5* (positive control) in the shoots of Arabidopsis Col-0 (wild type) seedlings are shown. Plants were grown in plates containing half-strength Murashige and Skoog (1/2 MS)-agar medium for 7 days and then under N-deficient conditions (0.3 mM N) for the indicated time periods. Transcript levels of each gene were normalized against the transcript levels of *ACTIN2* (*ACT2*) and then against the value obtained from samples at time zero. Data represent mean ± standard deviation (SD) of four biological replicates.

Under N deficiency stress, the timing of chlorophyll pigment degradation is consistent with that of photosystem protein degradation, and is accompanied by the loss of green color. The degradation of chlorophyll pigments occurs in two distinct phases. The first phase is associated with the degradation of chlorophyll pigments and their intermediates in the chloroplast, while the second phase involves the translocation of colorless chlorophyll catabolites from the chloroplast to the vacuole (Kuai et al., 2018). In the first phase, the degradation of chlorophyll molecules is catalyzed by at least seven enzymes. This catabolic process starts with the conversion of chlorophyll *b* to 7-hydroxymethyl chlorophyll *a* by two chlorophyll *b* reductase isoforms, NON-YELLOW COLORING 1 (NYC1) and NYC1-LIKE (NOL) (Kusaba et al., 2007; Horie et al., 2009), and is followed by the conversion of 7-hydroxymethyl chlorophyll *a* to chlorophyll *a* by 7-HYDROXYMETHYL CHLOROPHYLL *a* REDUCTASE (HCAR) (Meguro et al., 2011). The dechelation of magnesium from chlorophyll *a* is catalyzed by a magnesium-dechelatase, NON YELLOWING 1 (NYE1)/STAY-GREEN1 (SGR1) (Shimoda et al., 2016), and the product of this reaction (pheophytin *a*) is then dephytylated by PHEOPHYTINASE (PPH) to form pheophorbide *a* (Schelbert et al., 2009). Subsequently, the chlorin macrocycle of pheophorbide *a* is oxygenolytically opened by PHEOPHORBIDE *a* OXYGENASE (PAO) (Pruzinská et al., 2003) to form red chlorophyll catabolite (RCC), which is then reduced to a non-phototoxic chlorophyll catabolite, primary fluorescent chlorophyll catabolite (pFCC), by RCC REDUCTASE (RCCR) (Pruzinská et al., 2007). These seven chlorophyll catabolic enzymes physically interact with each other and with LHCII (Sakuraba et al., 2012b, Sakuraba et al., 2013), indicating that these chlorophyll catabolic enzymes form a multi-protein, and potentially highly dynamic, complex for cellular detoxification during leaf senescence (**Figure 1** **)**. During dark-induced and developmental leaf senescence, the expression levels of genes encoding some of the chlorophyll catabolic enzymes increase rapidly (Schelbert et al., 2009). In Arabidopsis, the expression levels of *NYC1*, *HCAR*, *NYE1/SGR1*, *PPH*, and *PAO* increased significantly under N deficiency stress (**Figure 2**), which implies that these enzymes are involved in chlorophyll degradation during N starvation-induced leaf senescence.

### Amino acid metabolism during N deficiency-induced N remobilization

Gln and Asn residues are the major carriers of N in the phloem sap of higher plants (Hayashi and Chino, 1990), and thus their synthesis in source organs is considerably important for N remobilization. The glutamine synthetase/glutamine-2-oxoglutarate aminotransferase (GS/GOGAT) cycle is considered to be the primary route of N assimilation in higher plants (Xu et al., 2012). In this cycle, GS catalyzes the synthesis of Gln from ammonium and glutamate (Glu) (Lam et al., 2003), while glutamate synthetase GOGAT catalyzes the transfer of the amide group of Gln to 2-oxoglutarate (2-OG) to synthesize two molecules of Glu (Suzuki and Knaff, 2005). On the other hand, asparagine synthetase (ASN) catalyzes the conversion from Glu to Asp to form Asn, and plays a vital role in N assimilation, remobilization, and allocation within the plant (Gaufichon et al., 2010).

Enzymes associated with the accumulation of Gln and Asn play important roles in the regulation of leaf senescence. The Arabidopsis genome harbors three *ASN* genes (*AtASN1–3*), among which *AtASN2* is the most highly expressed in the shoots (Gaufichon et al., 2010). The *atasn2* knockout mutants exhibit delayed leaf yellowing and relatively low *SAG12* expression compared with the WT during developmental senescence (Gaufichon et al., 2013). Similarly, in rice (*Oryza sativa* L.), knockout mutation of *OsASN1*, encoding one of the two ASN isoforms that plays a major role in Asn synthesis in roots (Ohashi et al., 2015), leads to the stay-green phenotype during developmental senescence (Lee et al., 2020). Delayed leaf yellowing observed in *asn* knockout mutants is probably caused by the decline in Asn synthesis, which likely leads to the stabilization of chlorophyll and other N-containing compounds in leaves. On the other hand, a rice knockout mutant of the gene encoding ferredoxin-dependent GOGAT (*OsFd-GOGAT*), which accumulates 10-fold more Gln than the WT, exhibited accelerated leaf yellowing during the reproductive phase, probably because of the promotion of N remobilization from old leaves to young leaves and other sink organs (Zeng et al., 2017).

The export of amino acids from older leaves is an important process for N remobilization under N deficiency stress. In poplar (*Populus trichocarpa*), the Gln content of senescing leaves dramatically increased, and a cationic amino acid transporter, Pt-CAT11, played an important role in the transfer of Gln during the senescence process (Couturier et al., 2010). In rice, overexpression lines of *OsAAP3* exhibited accelerated leaf yellowing phenotype, whereas RNA interference (RNAi) lines of *OsAAP3* exhibited delayed leaf yellowing (Wei et al., 2021). Higher plants possess a number of amino acid transporters (Yao et al., 2020); however, their functions in N remobilization during N deficiency stress remain unknown.

### Roles of nitrate transporters in N remobilization

Recent studies in Arabidopsis showed that NRTs play a critical role in the regulation of N starvation-induced leaf senescence and N remobilization. The Arabidopsis genome possesses 53 and 7 genes encoding Nitrate Transporter 1 (NRT1)/Peptide Transporter (PTR) and NRT2 proteins, respectively (Tsay et al., 2007). NRT2 proteins function as high-affinity nitrate transporters, while most NRT1/PTR family proteins have been functionally characterized as low-affinity nitrate transporters (Krapp et al., 2014; Forde, 2000).

Among the NRT1/PTR family proteins, NRT1.1 is considered as a unique protein, since it acts as a dual affinity transporter that can facilitate nitrate uptake at concentrations ranging from micromolar to millimolar (Liu et al., 1999). Genome-wide association study (GWAS) of Arabidopsis accessions suggested a significant association between differences in N starvation-induced leaf yellowing and *NRT1.1* sequence diversity (Sakuraba et al., 2021b). Indeed, *nrt1.1* knockout mutant, *chl1-5* exhibited accelerated leaf yellowing, while transgenic *NRT1.1*-overexpressing (*NRT1.1*-OX) plants retained greenness under N-deficient conditions (Sakuraba et al., 2021b). Furthermore, grafted seedlings generated using *NRT1.1*-OX scion and WT (Col-0) rootstock exhibited delayed leaf yellowing under the N-deficient conditions; however, such a delayed leaf yellowing phenotype was not conserved when chimeras were generated by grafting WT (Col-0) on *NRT1.1*-OX rootstock (Sakuraba et al., 2021b), indicating that the enhanced expression of *NRT1.1* in aboveground plant parts negatively regulates N starvation-induced leaf yellowing.

Arabidopsis NRT1.5, which is also classified into the NRT1/PTR family, is involved in the nitrate loading of the xylem (Lin et al., 2008). The expression of *NRT1.5* is highly upregulated during leaf senescence (van der Graaff et al., 2006). Leaves of *nrt1.5* knockout (*nrt1.5*-KO) mutant plants turned yellow at a rate comparable with those of WT plants when grown under low N (i.e., low nitrate) conditions; however, leaves of the *nrt1.5*-KO mutant turned yellow much earlier than those of WT plants under low N conditions, when the only N source was ammonium or amino acids (Meng et al., 2016), indicating that accelerated leaf yellowing in *nrt1.5*-KO mutants is caused specifically by the nitrate starvation. Furthermore, the accelerated leaf yellowing phenotype of *nrt1.5*-KO mutants was diminished by supplementation with 10 mM foliar potassium (K). Additionally, K supply during nitrate starvation suppressed the expression of several genes associated with K acquisition, including *HIGH-AFFINITY K^+^ TRANSPORTER 5* (*HAK5*), which encodes a major transporter that contributes to K uptake by roots (Gierth et al., 2005), and *RAP2.11*, which encodes a transcriptional regulator of *HAK5* (Kim et al., 2012), in *nrt1.5*-KO mutants (Meng et al., 2016). K supplementation also inhibited stress-induced yellowing of flag leaves in barley (*Hordeum vulgare* L.) (Hosseini et al., 2016). These findings suggest that NRT1.5 suppresses nitrate starvation-induced leaf senescence by modulating the K level.

Arabidopsis *NRT1.7*, which encodes a low-affinity nitrate transporter, is highly expressed in the phloem tissues of leaves, and its expression increases as leaves age (Fan et al., 2009). The *nrt1.7*-KO mutants highly retained nitrate in older leaves, and exhibited severe growth defects and premature leaf yellowing phenotype compared with WT plants when grown under N deficiency stress (Fan et al., 2009). Other Arabidopsis NRT1 family members, including NRT1.11 and NRT1.12, are also involved in nitrate remobilization from older to younger leaves. Both *NRT1.11* and *NRT1.12* are highly expressed in fully expanded rosette leaves, and the *nrt1.11 nrt1.12* double mutant exhibits lower nitrate content than the WT (Hsu and Tsay, 2013). However, the role of *NRT1.11* and *NRT1.12* in the response to N deficiency stress remains unknown. Considering the functions of NRT1 family proteins in the regulation of N starvation-induced leaf senescence, impaired nitrate remobilization in specific tissues may contribute to overall N deficiency, leading to the promotion of leaf senescence.

On the contrary, the involvement of NRT2 family proteins in the regulation of N deficiency-induced leaf senescence has not yet been elucidated. However, the expression levels of four Arabidopsis *NRT2* genes (*NRT2.1*, *NRT2.2*, *NRT2.4*, and *NRT2.5*) significantly increase during N deficiency (Lezhneva et al., 2014). While *NRT2.1*, *NRT2.2*, and *NRT2.4* are dominantly expressed in roots, *NRT2.5* is also expressed in shoots and is upregulated during N deficiency (Lezhneva et al., 2014). Thus, it is likely that some of the NRT2 family proteins also play important roles in the regulation of N starvation-induced leaf senescence.

## Transcriptional regulatory network of N starvation-induced leaf senescence

To cope with N deficiency stress, plants increase the capacity of N acquisition by enhancing the expression of genes associated with high-affinity transport systems for nitrate and ammonium (Kiba and Krapp, 2016). When these adaptations are not enough to provide a sufficient N supply, however, plants are forced to respond with further adaptive transcriptomic strategies for the remobilization of N to complete their life cycle.

In the last two decades, a number of leaf senescence-associated transcription factors have been identified and characterized in Arabidopsis and other plant species (Woo et al., 2019; Guo et al., 2021), which has greatly expanded our knowledge of the transcriptional regulatory network of leaf senescence. The functions of these senescence-associated transcription factors have been studied mostly during developmental progression-induced natural senescence or dark-induced leaf senescence (Guo and Gan, 2006; Kim et al., 2009; Kim et al., 2013; Sakuraba et al., 2014b). While dark-induced leaf senescence is known to be partially caused by the decline in N metabolism (Watanabe et al., 2013), the transcriptional regulatory network involved in this process remains unclear. Section 4 summarizes the recently reported transcriptomic changes that occur during N deficiency and the key regulatory modules involved in N starvation-induced leaf senescence.

### Transcriptomic changes in plants during N deficiency

Changes of the transcriptome of Arabidopsis plants during N starvation have been investigated using several different experimental approaches. Scheible et al. (2004) used seedlings grown initially in N-replete liquid medium and then in N-limited liquid medium for several days for DNA microarray analysis (Scheible et al., 2004). Bi et al. (2007) used the leaves of 3-week-old Arabidopsis plants grown hydroponically under mild and severe N deficiency (1 and 0.3 mM N, respectively) for DNA microarray analysis (Bi et al., 2007). Balazadeh et al. (2014) used leaves collected from Arabidopsis plants initially grown under N-sufficient conditions for 19 days and then grown under N-free conditions for several additional days to perform DNA microarray analysis (Balazadeh et al., 2014). Although Krapp et al. (2011) used a similar approach for growing Arabidopsis plants as described above, these plants were grown under short-day (8 h light/16 h dark) photoperiod (Krapp et al., 2011).

In the transcriptome data obtained from these studies, several sets of genes were commonly up- or downregulated. For instance, several genes associated with anthocyanin biosynthesis, including *CHALCONE SYNTHASE* (*CHS*), *DIHYDROFLAVONOL 4-REDUCTASE* (*DFR*), *PRODUCTION OF ANTHOCYANIN PIGMENT 1* (*PAP1*), and *PAP2*, were significantly upregulated in the leaves of plants exposed to N starvation. On the other hand, genes associated with cell wall organization, including *EXPANSIN A1* (*EXPA1*) and *EXPA8*, as well as those associated with photosynthesis and chlorophyll synthesis, including *HEMA1* and *HEME2*, were downregulated in shoots under N starvation (Scheible et al., 2004; Krapp et al., 2011). These observations were expected, since anthocyanin accumulation in shoots increases while shoot growth rate and photosynthetic activity decline under N starvation.

In addition, a number of senescence-associated genes were differentially regulated under N starvation. In the DNA microarray analysis performed by Balazadeh et al. (2014), more than half of the N starvation-induced genes were upregulated during developmental senescence upregulated genes, including *SAG12*, *SAG13*, and *ANAC029*/*NAC-LIKE, ACTIVATED BY AP3/PI* (*NAP*) (Buchanan-Wollaston et al., 2005; Breeze et al., 2011; Balazadeh et al., 2014). Upregulation of *NAP* under N starvation has also been reported in other transcriptome analyses (Scheible et al., 2004; Bi et al., 2007; Krapp et al., 2011; Balazadeh et al., 2014). NAP is classified into NO APICAL MERISTEM/ATAF1,2/CUP-SHAPED COTYLEDON (NAC) transcription factor family, and acts as an enhancer of developmental senescence and dark-induced leaf senescence by directly upregulating the expression of senescence-associated genes, including *SAG113* and *ABSCISIC ALDEHYDE OXIDASE 3* (*AAO3*) (Guo and Gan, 2006; Lei et al., 2020). In addition, the senescence-inducible gene *DUF581* was upregulated under N starvation in all transcriptome analyses described above (Krapp et al., 2011; Balazadeh et al., 2014).

Transcriptomic changes under N starvation-induced leaf senescence in the oilseed rape (*Brassica napus*) have been investigated using the cultivars that exhibit different responses to N starvation: NPZ-1 and Apex cultivars exhibit stay-green, while NPZ-2 and Capitol exhibit accelerated leaf yellowing under N deficient conditions (Schulte auf’m Erley et al., 2007; Koeslin-Findeklee et al., 2015a). As in the case in Arabidopsis, N deficiency in the leaves of oilseed rape also induced several senescence-associated genes, including *NAP* and *SGR1*, and some of these senescence-associated genes were highly expressed in the early senescing NPZ-2 and Capitol cultivars than in the stay-green NPZ-1 and Apex cultivars (Koeslin-Findeklee et al., 2015a). Moreover, biologically inactive cytokinins highly accumulated in the early senescing NPZ-1 and Apex cultivars, probably due to the altered expression of genes involved in the cytokinin homeostasis, including *CYTOKININ OXIDASE/DEHYDROGENASE2* (*CKX2*) (Koeslin-Findeklee et al., 2015b). Since the cytokinins are senescence-delaying phytohormones (Gan and Amasino, 1995), the homeostasis of biologically active cytokinins may be one of the predominant factors for the differences in N starvation-induced leaf senescence among cultivars of oilseed rape.

### Roles of NAC transcription factors in the promotion of N deficiency-induced leaf senescence

**Figure 3:**
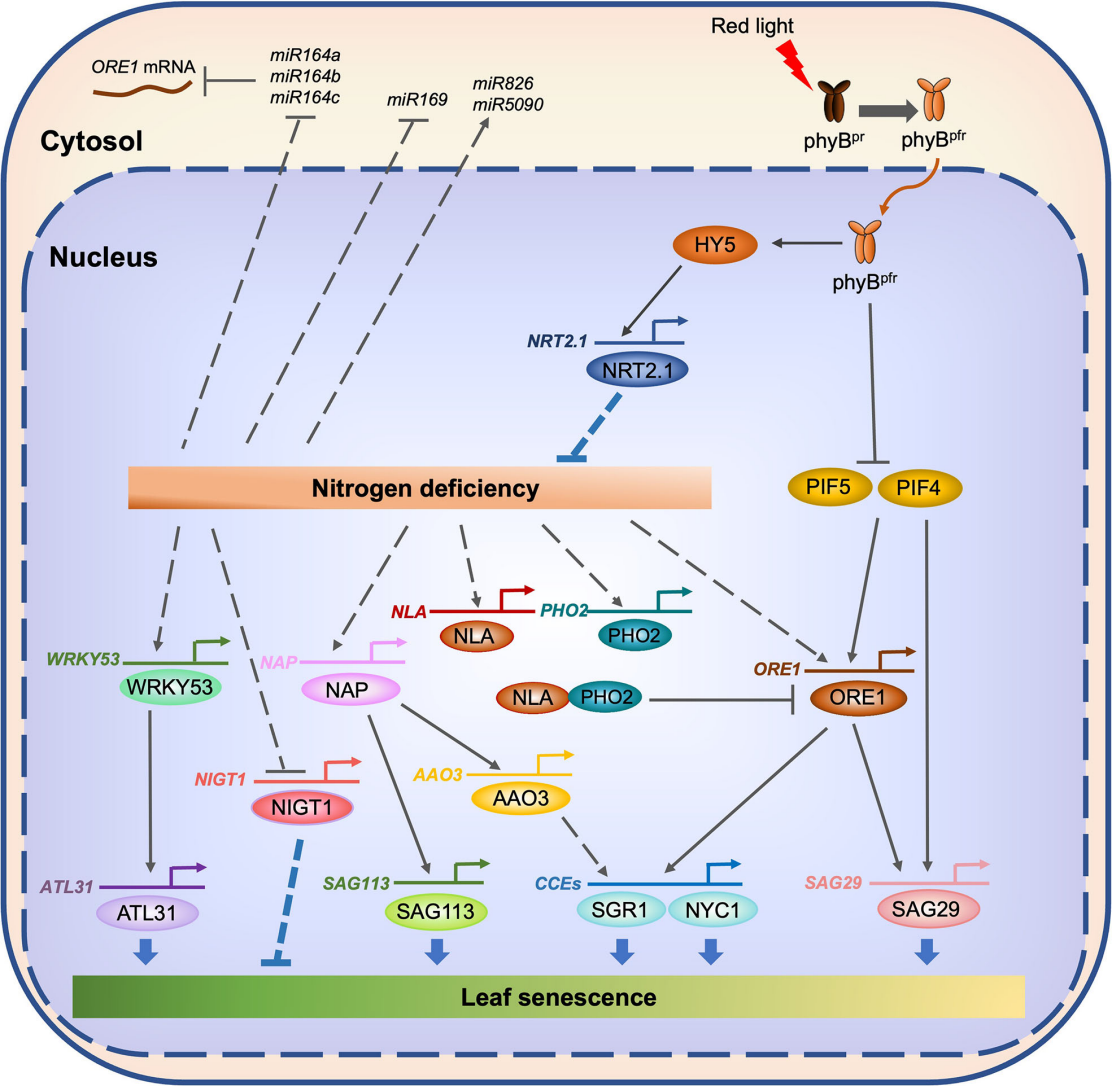
Transcriptional regulatory network of N starvation-induced leaf senescence in Arabidopsis. Under N-deficient conditions, the expression of several senescence-associated genes, including *WRKY53*, *NAP*, and *ORE1*, is enhanced. On the other hand, N deficiency downregulates the expression of *miR164*, which destabilizes *ORE1* mRNA, leading to a further increase of the accumulation of ORE1 protein. WRKY53 directly enhances the expression of *ATL31*, a key regulator of high C/low N-induced leaf senescence. NAP directly enhances the expression of *SAG113* and *AAO3*. ORE1 directly enhances the expression of *SAG29* and genes encoding chlorophyll catabolic enzymes (CCEs) including *SGR1* and *NYC1*. On the other hand, the expression of two genes associated with N deficiency responses, *NLA* and *PHO2*, is also enhanced. The NLA and PHO2 proteins promote the ubiquitination of ORE1, which leads to the degradation of ORE1, thus allowing the maintenance of a proper ORE1 protein level during N starvation-induced leaf senescence. phyB-mediated red light signaling may involve the suppression of N starvation-induced leaf senescence. Under the red light, the active Pfr form of phyB moves from cytosol to the nucleus. Under the downstream of phyB, HY5 directly activates the expression of genes associated with N acquisition, including *NRT2.1*. On the other hand, phyB promotes the proteasomal degradation of PIF4 and PIF5, which directly activate the expression of several senescence associated genes, including *ORE1* and *SAG29*. Solid lines indicate direct regulation, while dotted lines indicate indirect regulation.

The NAC family is one of the plant-specific transcription factor families (Riechmann et al., 2000). To date, a number of NAC transcription factors have been identified in Arabidopsis and other plant species as key regulators of leaf senescence (Sakuraba et al., 2015; Kim et al., 2016; Pimenta et al., 2016; Sakuraba et al., 2020). Among the senescence-associated NAC transcription factors in Arabidopsis, the functions of ORESARA1 (ORE1)/ANAC092 have been widely studied. A number of studies revealed that ORE1 acts as a central regulator of both developmental senescence and dark-induced leaf senescence. Additionally, the regulatory cascades for the induction of *ORE1* (Kim et al., 2009; Sakuraba et al., 2014b; Kim et al., 2018; Yu et al., 2021) and its downstream target genes (Matallana-Ramirez et al., 2013; Qiu et al., 2015) have been identified. A recent study showed that ORE1 also acts as a key regulator of N starvation-induced leaf senescence. On N-deficient growth medium, leaves of the *ore1* knockout mutant turned yellow much faster than those of the WT, while the leaves of *ORE1* overexpressors (*ORE1*-OX) retained their green color (Park et al., 2018). Under N deficiency, the transcript level of *ORE1* was elevated 15–20-fold. In addition, the mRNA levels of *PHOSPHATE 2* (*PHO2*), encoding a ubiquitin-conjugating E2 enzyme (Bari et al., 2006), and *NITROGEN LIMITATION ADAPTATION* (*NLA*), encoding an E3 ubiquitin ligase that acts together with PHO2 (Park et al., 2014), were also elevated during N deficiency (Park et al., 2018). ORE1 interacts with NLA in the nucleus and then destabilized through polyubiquitination by the NLA–PHO2 module (Park et al., 2018). On the other hand, ubiquitin-specific protease 12 (UBP12) and UBP13, which act redundantly in the de-ubiquitination of target proteins (Derkacheva et al., 2016; Jeong et al., 2017), remove the ubiquitin moieties from the polyubiquitinated ORE1 protein, restoring its stable state. Thus, these two deubiquitinases counteract the effect of the NLA–PHO2 module during ORE1-mediated N starvation-induced leaf senescence (**Figure 3**).

In apple (*Malus domestica*), MdNAC4 participates in N starvation-induced leaf senescence. When grown under N-deficient conditions, the leaves of *MdNAC4*-overexpressing plants turned yellow faster than those of the WT, whereas the leaves of *MdNAC4*-antisense plants retained greenness (Wen et al., 2022). MdNAC4 was shown to directly enhance the expression of two genes encoding chlorophyll catabolic enzymes, *MdNYC1* and *MdPAO* (Wen et al., 2022). Additionally, MdNAC4 physically interacts with PSEUDO-RESPONSE REGULATOR2 (MdAPRR2), which enhances the expression of genes encoding chlorophyll biosynthesis enzymes, including MdHEMA, MdCHLI, and MdCHLM, to inhibit the activity of MdAPRR2 (Wen et al., 2022). Thus, the interaction between MdNAC4 and MdAPRR2 appears to be a balancing mechanism for regulating N starvation-induced leaf senescence in apple.

### Roles of WRKY53 and ATL31 in the regulation of high carbon (C)/low N-induced leaf senescence

The carbon (C) status of plants affects N deficiency-induced leaf senescence. The rosette leaves of Arabidopsis WT (Col-0) plants turned yellow under high C and low N conditions (780 ppm CO_2_ and 0.3 mM N) but not under low C and low N conditions (280 ppm CO_2_ and 0.3 mM N), even at the same growth stage (Aoyama et al., 2014), indicating that the C status of plants is one of the key determinants of N starvation-induced leaf senescence. ARABIDOPSIS TOXICOS EN LEVADURA 31 (ATL31), a RING-type ubiquitin ligase, regulates the balance between C and N availability in Arabidopsis (Sato et al., 2009). Under high C/low N conditions, the leaves of *atl31*-KO plants turned yellow faster than those of WT plants, while the leaves of *ATL31*-OX plants retained greenness much longer (Aoyama et al., 2014), indicating that ATL31 acts as a negative regulator of high C/low N-induced leaf senescence. The transcript level of *ATL31* significantly increases under high C/low N conditions, similar to the expression pattern of the Arabidopsis *WRKY53* gene, which encodes a senescence-associated WRKY transcription factor (Miao et al., 2004; Miao and Zentgraf, 2007). WRKY53 was shown to activate the promoter of *ATL31* (Aoyama et al., 2014), indicating that WRKY53 acts as an enhancer for the induction of *ATL31* under high C/low N conditions (**Figure 3**). WRKY53 is one of the most widely studied senescence-associated transcription factors in Arabidopsis, and several key factors in the regulation of *WRKY53* expression and protein activity have been identified and characterized (Miao et al., 2013; Zentgraf and Doll, 2019; Doll et al., 2020). Additionally, the downstream targets of WRKY53 have been identified (Miao et al., 2004). While Aoyama et al. (2014) showed that WRKY53 enhances the expression of *ATL31*, it is still not clear how WRKY53 affects the promotion of N starvation-induced leaf senescence. Investigation of the effects of knockout mutation and overexpression of *WRKY53* on the promotion of N deficiency-induced leaf senescence is necessary for understanding the significance of the WRKY53–ATL31 regulatory module in N starvation-induced leaf senescence.

### Possible involvement of phytochrome B-mediated red light signal in the regulation of N starvation-induced leaf senescence

While red light has long been considered to delay leaf senescence (Pfeiffer and Kleudgen, 1980; Tucker, 1981), the molecular mechanisms underlying red light signaling-mediated regulation of leaf senescence were partially revealed only in the last decade, especially in the model plant Arabidopsis. Among five phytochromes in Arabidopsis, namely phyA, phyB, phyC, phyD, and phyE (Sharrock and Clack, 2002), phyA plays a major role in the far-red light response (Reed et al., 1994). On the other hand, phyB–phyE are involved in the red/far-red low-fluence response *via* the reversible transition between the red-light-absorbing biologically inactive Pr form and the far-red-light-absorbing biological active Pfr form (Li et al., 2011). Among five Arabidopsis phytochromes, phyB is involved in the regulation of dark-induced leaf senescence; two *phyB* knockout mutants, namely *phyB-9* and *phyB-10*, exhibited accelerated leaf yellowing, while *phyB* overexpressors highly retained greenness after the dark incubation (Sakuraba et al., 2014b). PHYTOCHROME INTERACTING FACTOR4 (PIF4) and PIF5 act downstream of phyB, and promote both age-dependent and dark-induced leaf senescence by directly enhancing the expression of senescence-associated genes, including *ORE1*, *ETHYLENE INSENSITIVE 3* (*EIN3*), *ABA INSENSITIVE 5* (*ABI5*), *ENHANCED EM LEVEL* (*EEL*), *SGR1*, and *SAG29* (Sakuraba et al., 2014b; Song et al., 2014; Zhang et al., 2015; Sakuraba et al., 2017). In rice, phyB-mediated red light signaling is also involved in the promotion of dark-induced leaf senescence; the *osphyB* T-DNA insertion knockout mutant exhibited accelerated leaf yellowing during dark-induced leaf senescence (Piao et al., 2015). In addition, RNAi of *PIF4* in tomato (*Solanum lycopersicum* L.) delayed greenness during developmental leaf senescence (Rosado et al., 2019), indicating that phyB/PIF-mediated red-light signaling acts as a key regulatory module of dark-induced and developmental leaf senescence in many plant species.

While the role of phyB-mediated red light signaling in the regulation of dark-induced leaf senescence has been widely studied, this mechanism has also been shown to affect leaf senescence under light. Detached leaves of the *osphyB* knockout mutant turned yellow faster than those of WT plants when incubated in N-free liquid medium, and the accelerated leaf yellowing phenotype of *osphyB* leaves was recovered by the supplementation with N sources such as potassium nitrate (KNO_3_) and ammonium nitrate (NH_4_NO_3_) (Piao et al., 2015), indicating that N availability is one of the key determinants for OsphyB-mediated regulation of leaf senescence under light conditions.

In Arabidopsis, phyB-mediated red-light signaling is involved in the promotion of phosphate ( $PO_{4}^{3-}$ ) uptake *via* roots by enhancing the expression of high-affinity phosphate transporter genes, including *PHOSPHATE TRANSPORTER1;1* (*PHT1;1*) (Sakuraba et al., 2018). On the other hand, the expression of ammonium transporter genes, *AMT1;1*, *AMT1;2*, and *AMT2;1*, was upregulated in Arabidopsis seedlings grown under red-light illumination (Huang et al., 2015). Genome-wide chromatin immunoprecipitation sequencing (ChIP-seq) analyses showed that HY5, a positive regulator of phyB-mediated red-light signaling (Gangappa and Botto, 2016), as well as PIF4, directly bind to the promoters of genes associated with the uptake and assimilation of N (Lee et al., 2007; Oh et al., 2012). Moreover, upon the exposure of plants to light, HY5 is translocated from shoots to roots, where it directly enhances the expression of *NRT2.1* (Chen et al., 2016). Considering the involvement of phyB-mediated red-light signaling in the acquisition of nutrients in Arabidopsis, it is highly likely that phyB-mediated red-light signaling also plays important roles in the regulation of N starvation-induced leaf senescence.

### Involvement of NIGT1 transcription factors in the regulation of N starvation-induced leaf senescence

NITRATE-INDUCIBLE, GARP-TYPE TRANSCRIPTIONAL REPRESSOR1 (NIGT1) transcription factors act as negative regulators in nitrate inducible gene expression. In Arabidopsis, NIGT1 transcription factors directly repress *NRT2* genes and other N deficiency-inducible genes (Maeda et al., 2018; Kiba et al., 2018), and thus a gradual reduction in *NIGT1* transcript levels under N deficiency leads to the activation of N deficiency-inducible genes (Kiba et al., 2018).

Very recently, Tan et al. (2022) reported that *Malus domestica* HYPERSENSITIVE TO LOW PI-ELICITED PRIMARY ROOT SHORTENING1 (HRS1) HOMOLOG3 (*Md*HHO3), which is phylogenetically classified into NIGT1 family protein, directly represses *Malus domestica NRT2.1* (*MdNRT2.1*) transcript level, similar to Arabidopsis NIGT1s (Tan et al., 2022). Moreover, Arabidopsis and tobacco transgenic plants that overexpressing *MdHHO3* exhibited premature leaf yellowing phenotype, with the upregulation of senescence-associated genes, such as *NYC1*, *PAO*, and *SGR1*, under N deficiency (Tan et al., 2022), that NIGT1 transcription factors also act as a negative regulator in the N starvation-induced leaf senescence.

## Involvement of microRNAs in the regulation of N starvation-induced leaf senescence

Small RNAs, including microRNAs (miRNAs) and small interfering RNAs (siRNAs), are considered the key signaling molecules that regulate the expression of genes at the post-transcriptional level. In plants, miRNAs play important roles in the regulation of various environmental stress responses, including nutrient deficiency responses (Paul et al., 2015).

During N starvation, the accumulation of a number of miRNAs also changes in Arabidopsis and other plant species. Small RNA sequencing of Arabidopsis seedlings grown under N-sufficient and N-deficient conditions showed that the expression of more than 20 *miRNAs* significantly decreased, while that of several *miRNAs* increased (Liang et al., 2012). Among these miRNAs, *miR826* was strongly upregulated during N deficiency, and transgenic Arabidopsis plants overexpressing *miR826* or *miR5090*, which was identified from the complementary transcripts of *miR826*, exhibited better growth with delayed leaf yellowing under N-deficient conditions (He et al., 2014). On the other hand, the expression of *miR169* significantly decreased during N deficiency, and transgenic Arabidopsis plants overexpressing *miR169* exhibited accelerated leaf yellowing phenotype under N-deficient conditions (Zhao et al., 2011). *miR164s* are known to target the mRNA of *ORE1*, a central regulator of leaf senescence in Arabidopsis, to repress its expression at post-transcriptional level (Kim et al., 2009). During N deficiency, the expression levels of three *miR164s* (*miR164a*, *miR164b*, and *miR164c*) significantly decreased, while the *ORE1* transcript level was significantly elevated (Park et al., 2018), indicating that *miR164s* are involved in the suppression of N starvation-induced leaf senescence.

As described above, several N starvation-responsive miRNAs function in the regulation of N starvation-induced leaf senescence. Thus, it would be highly interesting to elucidate the roles of other N starvation-responsive miRNAs, as well as N starvation-responsive siRNAs, for further understanding of the regulatory mechanisms in N starvation-induced leaf senescence at the post-transcriptional level.

## Conclusion and perspectives

N starvation-induced leaf senescence is a highly complex process finely controlled by several regulatory factors at different levels. To date, numerous genes associated with N starvation-induced leaf senescence have been identified, mostly in Arabidopsis. However, many more genes are expected to be involved in the regulation of N starvation-induced leaf senescence and to form a highly complex regulatory network. In recent years, several studies have attempted to identify the genes associated with N deficiency responses using experimental approaches that employ big data, such as GWAS and gene co-expression analysis, providing new insights into the mechanisms of N deficiency responses. In the GWAS using the parameter of the reduction in chlorophyll content of 52 Arabidopsis accessions grown under N deficient conditions, several peaks potentially associated with N deficiency-induced leaf yellowing were identified, and several genes, including *NRT1.1*, *AGAMOUS-LIKE65* (*AGL65*), *ATP-BINDING CASSETTE G1* (*ABCG1*), and *INOSITOL 1,3,4-TRISPHOSPHATE 5/6-KINASE 3* (*ITPK3*), were found near the peaks (Sakuraba et al., 2021b). As described in section 3.4, the significance of NRT1.1 in the regulation of N starvation-induced leaf senescence has been demonstrated. To dissect the gene regulatory network and identify novel genes associated with N deficiency responses in rice, Ueda et al. (2020) performed gene co-expression analysis and machine learning-based pathway inference using the transcriptome data of rice seedlings exposed to N-sufficient and N-deficient conditions (Ueda et al., 2020). Based on the results, several transcription factors were predicted to function as key regulators of the gene regulatory networks involved in N deficiency responses. In addition, transcription factors identified based on gene co-expression analysis and machine learning-based pathway inference also included OsNAC2, which acts as an enhancer of leaf senescence by controlling the accumulation of abscisic acid (ABA) (Mao et al., 2017), and OsWRKY23, which is used as a marker gene of leaf senescence (Han et al., 2020). Therefore, the transcription factors identified Ueda et al. (2020) most likely include key regulators of N starvation-induced leaf senescence. Functional characterization of each gene identified by the analyses using big data will further reveal the regulatory networks of N starvation-induced leaf senescence.

Recent studies revealed the significance of peptide hormones in the regulation of leaf senescence and nutrient starvation responses. In Arabidopsis, the small secreted peptide CLAVATA3/ESR-RELATED 14 (CLE14) functions in the suppression of leaf senescence by regulating the accumulation of reactive oxygen species (Zhang et al., 2022a). In Arabidopsis, CLE42 also acts as a negative regulator of leaf senescence by suppressing the biosynthesis of ethylene (Zhang et al., 2022b). Moreover, CLE42 showed functional redundancy with CLE41 and CLE44 in the suppression of leaf senescence: *cle41 cle42 cle44* triple mutant exhibited a strong early senescence phenotype (Zhang et al., 2022b). On the other hand, the root-to-shoot mobile peptide hormones C-TERMINALLY ENCODED PEPRIDEs (CEPs) and two CEP receptors (CEPRs) mediates N acquisition response accompanied by N-deficiency symptom to adapt to fluctuations in local N availability (Tabata et al., 2014). The involvement of these peptide hormones in the regulation of N starvation-induced leaf senescence is not yet investigated, therefore, examining this possibility is important for a better understanding of the molecular mechanisms underlying N starvation-induced leaf senescence.

While the molecular mechanisms underlying N starvation-induced leaf senescence have been studied mostly in the model plant Arabidopsis, the knowledge gained from Arabidopsis must be applied to crop plants. In the last two decades, the genome of a variety of crop plants has been sequenced (Goff et al., 2002; Schmutz et al., 2010; Sato et al., 2012), thus enabling the systematic analysis of plant biological processes, including N starvation-induced leaf senescence, by comparative genomics. Furthermore, owing to the recent advent of the CRISPR/Cas9 technology, which allows the modification of genomes without leaving behind any trace of foreign DNA (Jyoti et al., 2019), it is now possible to generate crop plants capable of displaying enhanced growth and high yield under a N-limited environment by modulating the function of gene(s) associated with N starvation-induced leaf senescence.

## Author contributions

The author confirms being the sole contributor of this work and has approved it for publication.

## Funding

This work was supported by the Japan Society for the Promotion of Science KAKENHI (22K05368).

## Acknowledgments

I apologize to authors whose publications could not be cited because of space constraints.

## Conflict of interest

The author declares that the research was conducted in the absence of any commercial or financial relationships that could be construed as a potential conflict of interest.

## Publisher’s note

All claims expressed in this article are solely those of the authors and do not necessarily represent those of their affiliated organizations, or those of the publisher, the editors and the reviewers. Any product that may be evaluated in this article, or claim that may be made by its manufacturer, is not guaranteed or endorsed by the publisher.
